# Effect of External Electric Field Stress on Gliadin Protein Conformation

**DOI:** 10.3390/proteomes1020025

**Published:** 2013-07-04

**Authors:** Ashutosh Singh, Shirin Munshi, Vijaya Raghavan

**Affiliations:** 1 Department of Bioresource Engineering, McGill University, 21111, Rue Lakeshore, Ste-Anne-de-Bellevue, QC H9X 3V9, Canada; E-Mail: vijaya.raghavan@mcgill.ca; 2 School of Dietetics and Human Nutrition, McGill University, 21111, Rue Lakeshore, Ste-Anne-de-Bellevue, QC H9X 3V9, Canada; E-Mail: shirin.munshi@mail.mcgill.ca

**Keywords:** molecular dynamic modeling, gliadin protein, root mean square deviation, radius of gyration, electric field, hydrogen bonds, food processing

## Abstract

A molecular dynamic (MD) modeling approach was applied to evaluate the effect of external electric field on gliadin protein structure and surface properties. Static electric field strengths of 0.001 V/nm and 0.002 V/nm induced conformational changes in the protein but had no significant effect on its surface properties. The study of hydrogen bond evolution during the course of simulation revealed that the root mean square deviation, radius of gyration and secondary structure formation, all depend significantly on the number hydrogen bonds formed. This study demonstrated that it is necessary to gain insight into protein dynamics under external electric field stress, in order to develop the novel food processing techniques that can be potentially used to reduce or eradicate food allergens.

## 1. Introduction

Gluten is a storage protein found in wheat, it plays an important role in regulating its baking quality by determining the viscosity, cohesivity, water absorption and elasticity of the dough [1]. Gluten protein found in wheat can be divided into two major fractions based on their solubility in aqueous alcohol; gliadin (soluble) and glutenin (insoluble). They both are characterized by their high content of glutamine and proline amino acids [1]. The presence of these amino acids not only determine the physical property of wheat-based products but also plays an important role in patho-physiology of autoimmune disorder called *coeliac* disease. 

Coeliac disease can be defined as a permanent intolerance to certain cereal proteins such as gluten in wheat, hordeins in barley and secalins in rye. These proteins contain high percentage of proline and glutamine residues, but their content varies widely between cereals and so is their toxicity level. Uncontaminated Oat is considered to be a safer cereal for coeliac patients due to lower content of proline residue in its protein avenin [2]. Several attempts have been made to determine the role of cereal proteins in coeliac disease currently for patients suffering from this disease a strict gluten-free diet is often the recommended treatment [1,2,3,4,5]. 

The complexity of a gluten-free diet has led food industries to look for alternatives and develop technologies to reduce or completely remove the effect of gluten protein on human health. In recent years several novel food-processing techniques have been developed, such as high pressure processing, pulsed electric field processing and electrohydrodynamics [6], but their application into cereal industries is still at its nascent stage. In this study authors have tried to evaluate the applicability of high electric field processing on wheat protein and check its feasibility at larger scale. Molecular dynamic modeling has been used to provide an insight and predict the effect of high electric field on gliadin protein conformation. It is known that protein conformation determines the functionality of the protein, any change in its conformation may lead to change in functionality and overall activity of the protein. Molecular modeling has been widely applied to study the changes in protein structure and related functionalities.

This study puts emphasis on effect of external electric field on the conformation of gliadin protein. Results obtained from the study provide an insight into what we can expect to happen, and since this study is the authors’ first attempt to check the viability and applicability of a novel food processing techniques using molecular dynamic simulation, only changes in conformation have been the focus point. Future work will explore deeper into the mechanism and changes in activity of gliadin protein.

## 2. Experimental

Molecular dynamic (MD) simulation of Gliadin protein was performed using molecular dynamic algorithms implements in Groningen machine for chemical structure (GROMACS) software package (version 4.5.4, Stockholm Center for Biomembrane Research, Stockholm, Sweden) [7,8]. Gliadin protein starting sequence configuration was obtained from Chain C of protein database bank (PDB) accession code 2NNA, which represents the structure of the MHC class II molecule HLA-DQ8 bound with a deamidated gluten peptide. The α-β gliadin peptide Chain C sequence (QQYPSGEGSFQPSQENPQ) (Table 1) had minimal to no defined secondary structures other than turns. All atoms CHARMM27 (Chemistry at Harvard Macromolecular Mechanics) force field was used in this study to provide the parameters and mathematical functions to describe the potential energy of all the atoms in the system. The protein configuration was enclosed in the center of a periodic cubic 3-Point (TIP3P) water box size 10.3 × 10.3 × 10.3 nm containing 33,832 water molecules to satisfy the minimum image convention. Two sodium ions were added to the system to neutralize it and later the solvated neutral system was energy minimized using steepest descent for 50,000 steps and equilibrated at constant temperature (NPT) and pressure (NPT) for 200 ps. MD simulation was carried out for 10 ns at constant temperature of 300 K and pressure of 1 bar maintained using Berendsen thermostat and barostat respectively [9,10]. For the temperature and pressure coupling was 0.1 ps and 2 ps respectively. A cut of 1 nm was used to limit the short-range non-bonded interactions, van der Waals interactions and long-range electrostatic interactions. PME algorithm was used with a grid spacing of 0.16 nm and time step during the simulation was set to 2 fs [10]. During the simulation the protein configuration was subjected to static electric field strength of 0.001 V/nm and 0.002 V/nm (Table 1). One MD simulation was run without electric field as a reference. We selected the electric field strength to simulate the achievable electric field intensity of 10 kV/cm and 20 kV/cm, commonly used for electrohydrodynamic drying (EHD) and pulsed electric field (PEF) processing of food [6].

**Table 1:** Primary sequence of α-β gliadin [Chain C of protein database bank (PDB): 2NNA].

	1	2	3	4	5	6	7	8	9
0	GLN	GLN	TYR	PRO	SER	GLY	GLU	GLY	SER
10	PHE	GLN	PRO	SER	GLN	GLU	ASN	PRO	GLN

Note: GLY: glycine; GLU: glutamic acid; GLN: glutamine; PRO: proline; ASN: asparagine; SER: serine; TYR: tyrosine; PHE: phenylalanine.

The conformational changes of the protein were represented using root mean square deviation (RMSD) and radius of gyration. The solvent accessible surface area of the protein was also analyzed to represent the changes in surface properties under the influence of the external electric field. Influence on hydrogen bonds evolution during the simulation was also analyzed, as they are needed for protein folding, binding to other proteins or enzymes and other processes. The STRIDE algorithm implemented in the visual molecular dynamics (VMD) software package was used to study the changes in the secondary structures of the protein [11]. 

## 3. Results and Discussion

### 3.1. Secondary Structure Analysis

**Figure 1:**
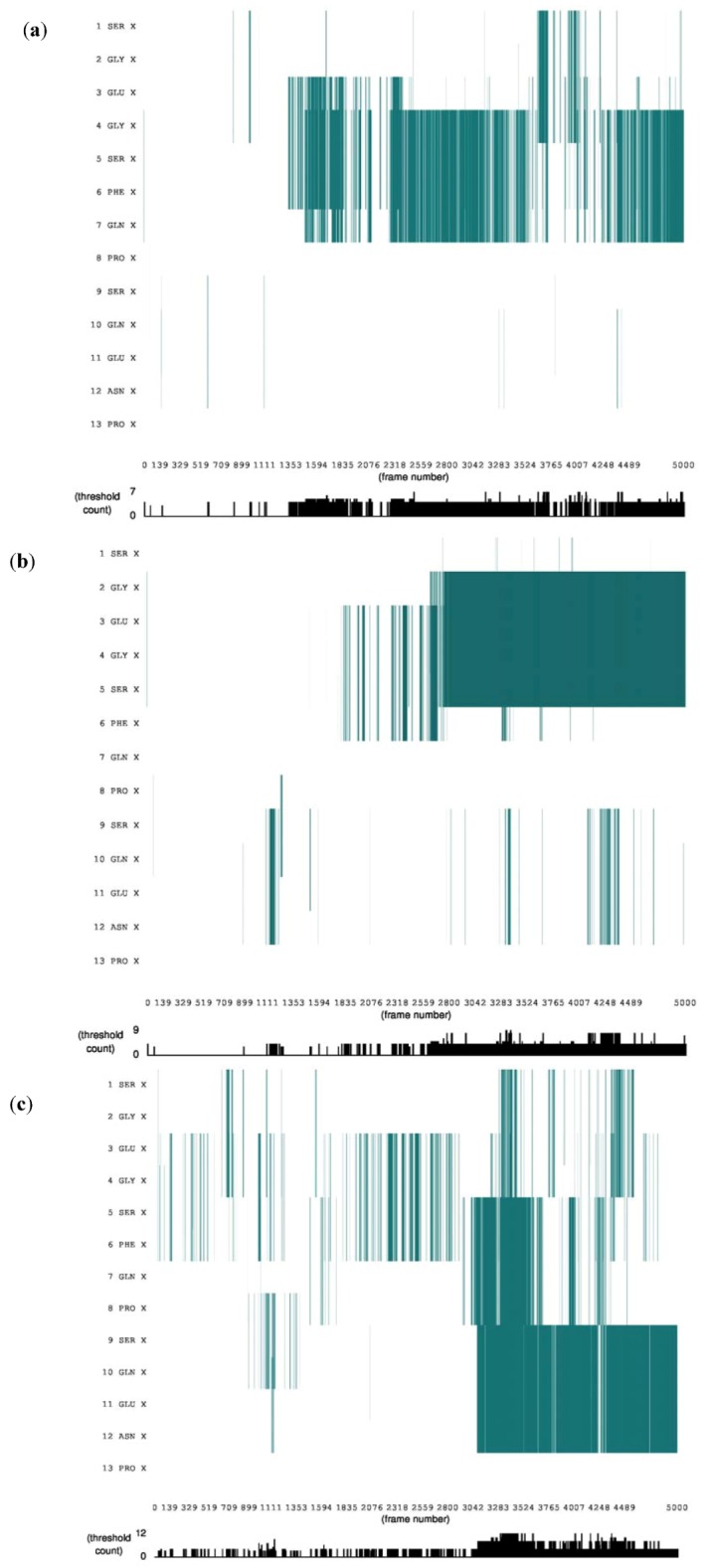
Stride evolution of secondary structures of gliadin protein under external electric field (**a**) Without external electric field; (**b**) Under electric field strength 0.001 V/nm; (**c**) Under electric field strength 0.002 V/nm. (Color code: Cyan denotes turn.)

Gluten protein comprises of about 50 protein components that can be classified into two major groups: monomeric gliadin and polymeric glutenin [8]. One subgroup of glutenin also called as high molecular weight (HMW) subunits of glutenin comprises of approximately 650–820 amino acid residues containing an extensive repetitive domain (480–680 residues) conferring a β-spiral (β-reverse turns) in a solution [8]. These subunits account for 8%–10% of the gluten protein, the rest consists of light molecular weight (LMW) subunits and α, β and γ gliadins [12]. The effect of external electric field on secondary structure of the gliadin protein was estimated using STRIDE algorithm implemented in VMD software package. The STRIDE algorithm [13], implemented in VMD [11] helps to simplify the analysis of tertiary conformational changes of a protein by assigning secondary structure type to each residues based on knowledge-based algorithm, which take into account hydrogen bond energy and statistically derived backbone torsional angle information. The protein conformation obtained from ChainC (PDB accession code: 2NNA) was subject to external electric field strength of 0.001 V/nm and 0.002 V/nm. The protein sequence obtained had no distinct secondary structures defined such as alpha helices and beta sheets, the only structure that was visible and analyzed were the turns (Figure 1). 

**Figure 2:**
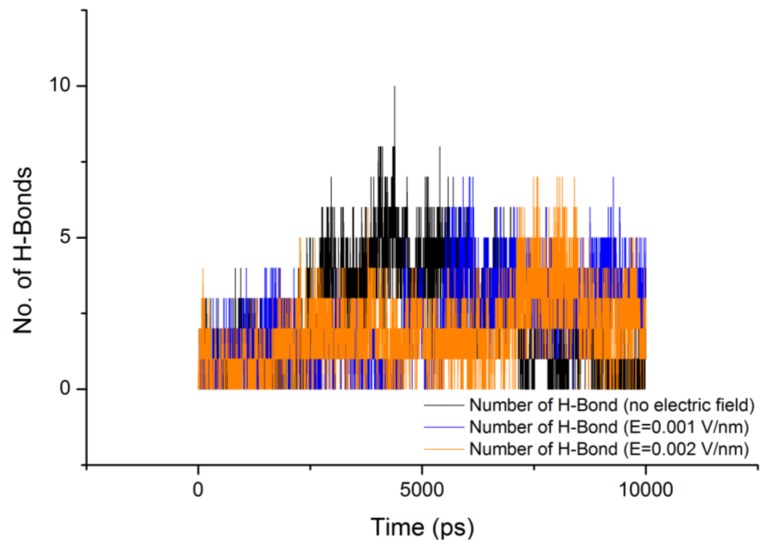
Evolution of hydrogen bonds during the course of simulation under the influence of external electric field.

There has been relatively little study of the conformational structure of gliadin protein. In 1988, Purcell *et al* [12] conducted a Fourier transform infrared spectroscopy (FTIR) study on secondary structures of wheat α- and ω-gliadin protein, they showed that α-helical structure may make a significant contribution to the secondary structure of α-gliadin, but they also suggested that the absorption spectrum which they observed near 1,654 cm^−1^ concluding presence of α-helical structure may be due to the presence of β-turns which have an absorption at 1,658 cm^−1^. They concluded that gliadin protein includes a significant proportion of β-turn structures. These observations made by Purcell *et al* [12] support our finding of no other secondary structure other than turns. Analysis of Figure 1 it show that as the protein configuration was subjected to electric field the number of protein amino acids involved in formation of the turn increased. This behavior can be attributed to glycine and proline residues present in the gliadin sequence, from Figure 2 suggests that during the course of the simulation both the number of hydrogen bonds and number of turns increased with time.

### 3.2. Dipole Moment Distribution

**Figure 3:**
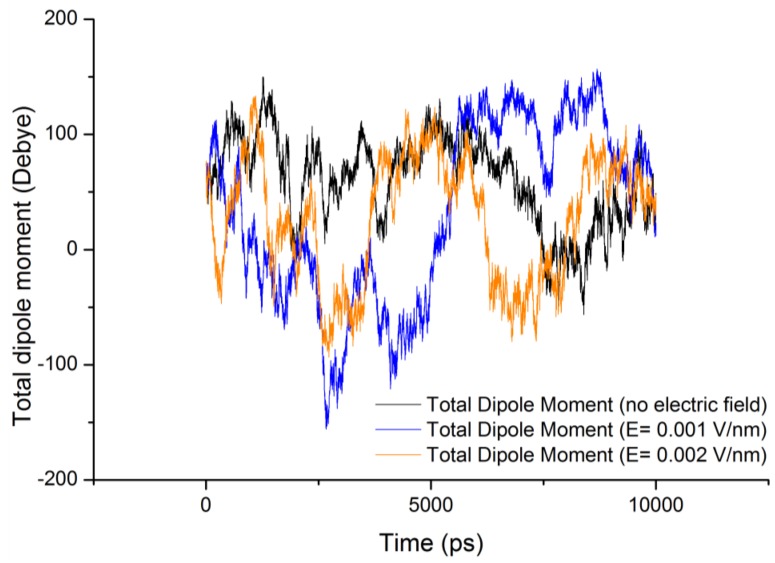
Total dipole moment of gliadin protein under the influence of an external electric field.

In general proteins possess electric dipole moment by virtue of their secondary structure conformations such as helices, sheets, turns, coils, *etc.* When an external electric field is applied, it induces an alignment change with respect to the direction of the applied field. The electric dipole of a protein is represented as [8]:

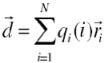
(1)
where 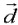 is the dipole, *q_i_* is the charge of the atom *i*, 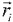 is the directional vector of each atom and *N* is the number of atoms. In our study, the electric field was applied in the *z-axis*. Depending on the strength of the applied field, a change in the total dipole moment of the gliadin protein was observed (Figure 3). 

From Figure 3 it can be observed that the total dipole moment of gliadin protein under the stress of external electric field varied significantly (Table 2). It is important to note that under the influence of electric field the protein structure tries to reorients itself in the direction of the field, but in the case of gliadin protein absence of any distinct secondary structures such as helices and beta-sheets, should have shown similar dipole moment as the reference (without electric field). However sharp changes in the total dipole moment were observed, which can be attributed to the electrical properties of the constituent amino acids trying to orient themselves in the direction of the field. For a protein with defined secondary structures such as helices, their dipole moment contributes to the binding of charges substrates or coenzymes at the termini and can also enhance the reaction rates [14].

**Table 2:** Backbone Root Mean Square Deviation (RMSD), radius of gyration and total dipole moment, averaged over 10 ns of simulation time.

Molecule	Electric field strength (V/nm)	RMSD average (nm)	Rg average (nm)	Total Dipole moment (Debye)
Gliadin protein	0	0.536 ± 0.131	0.942 ± 0.095	59.8 ± 38.66
Gliadin protein	0.001	0.461 ± 0.102	1.030 ± 0.065	33.3 ± 78.48
Gliadin protein	0.002	0.617 ± 0.137	0.911 ± 0.094	26.9 ± 53.41

### 3.3. Root Mean Square Deviation (RMSD)

Changes in the conformation of protein can be quantitatively measured by determining the RMSD of a protein. RMSD is estimated by comparing the structure of simulated protein during simulation with the reference. RMSD of a protein is represented as [8]:

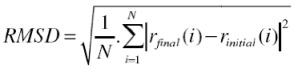
(2)
where *r_final_*(*i*) is the final coordinates of an atom *i*, and *r_initial_*(*i*) is the initial coordinate of the atom *i*, and *N *is the number of atoms.

**Figure 4:**
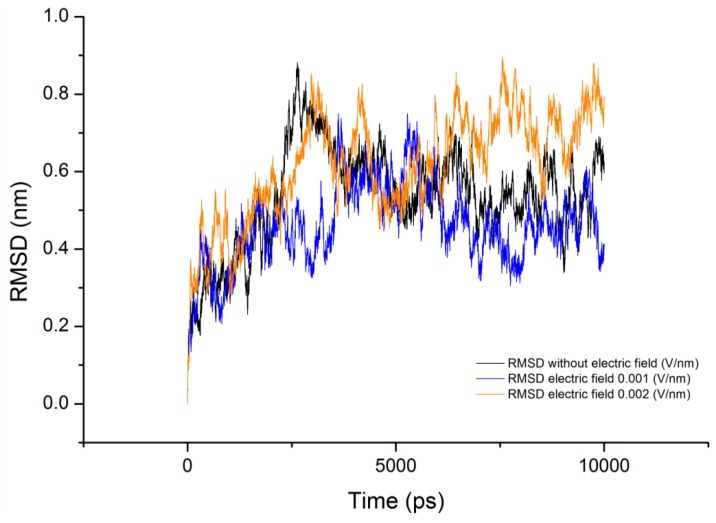
RMSD of the gliadin protein backbone under the influence of external electric field.

**Figure 5:**
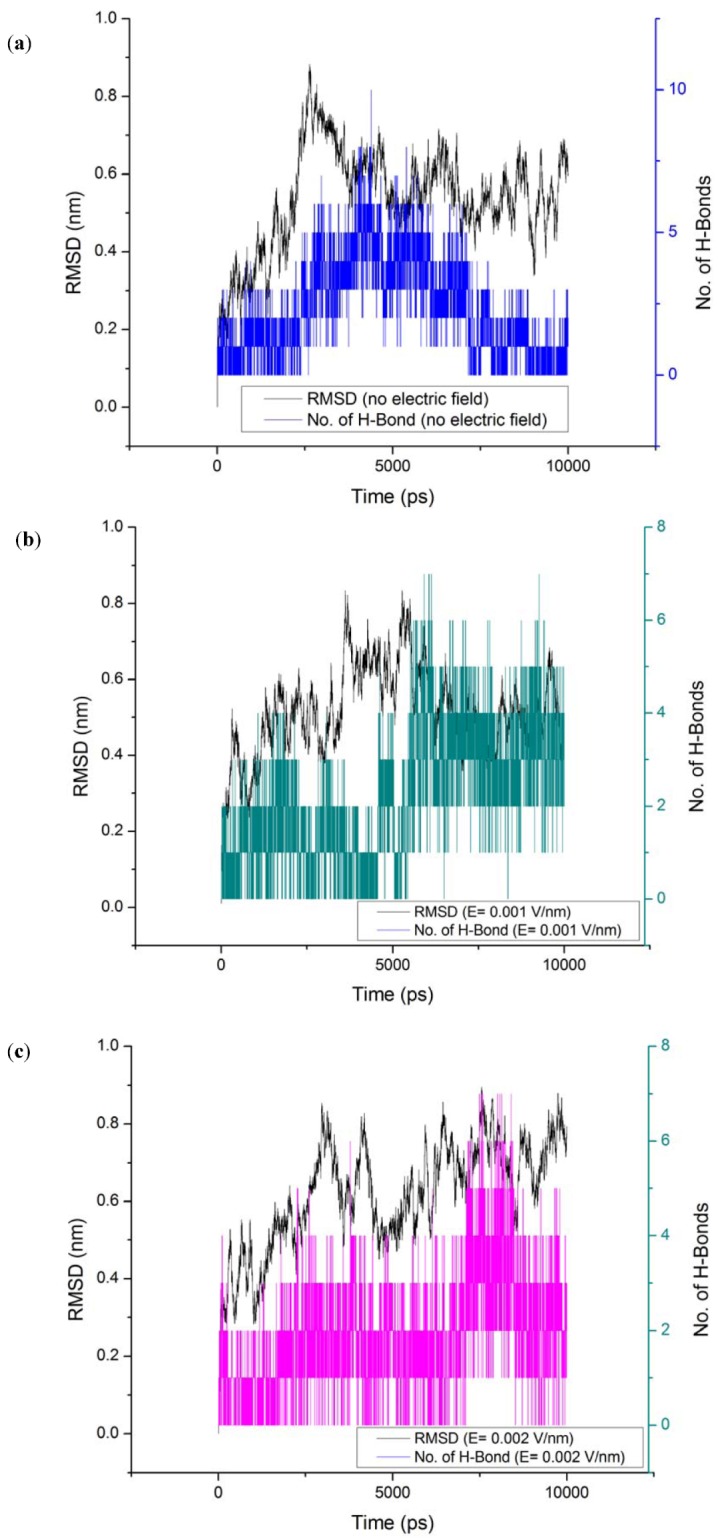
RMSD evolution with respect to number of hydrogen bonds; (**a**) without electric field (**b**) 0.001V/nm; (**c**) 0.002 V/nm.

The average value of RMSD obtained for the 10ns simulation is shown in Table 2. It can be observed that varied deviation can be observed in the RMSD of the protein under the influence of electric field. RMSD of the protein under field strength of 0.001 V/nm was lower compared to the reference and field strength of 0.002 V/nm (Figure 4). This variation in the protein RMSD can be attributed to the change in the orientation of the protein and involvement of amino acids in formation of turns via hydrogen bonds as observed in Figure 5. The trend analysis revealed that for reference simulation the RMSD value decreased with increase in number of hydrogen bonds (Figure 5). A similar trend was observed for simulation performed under electric field strength of 0.001 V/nm, but for electric field strength of 0.002 V/nm increase in number of hydrogen bond had no significant effect on the RMSD value. 

Hydrogen bonds play a significant role in stabilizing the secondary structures of proteins. The loss of internal hydrogen bonding between amino acids due to applied stress can cause destabilization of secondary structures and lead to protein unfolding [15]. Table 3 presents information about the number of hydrogen bonds (hbonds) formed during the course of simulation as a function of time under external electric field stress [16,17]. In this study, in order to be classified as a hydrogen bond, the donor-acceptor atom separation was set be no more than 3.5 Å. From Table 3 it can be observed that glutamine (GLN) and glutamic acid (GLU) were one of the major donor and acceptor of hbonds. In this study we analyzed the effect of external electric field stress on deamidated gliadin peptide. Deamidated gliadin peptide is produced by enzymatic action of tissue transglutaminase, which converts excess glutamines to glutamic acid and it is well known that cellular immunity to a deamidated α-β-gliadin is more severe than normal α-β-gliadin. Extensive immunological studies have been carried out on deamidated gliadins [1,18,19].

**Table 3:** Hydrogen bond formation between donor and acceptor amino acids and their occupancy (over 2%) during the course of simulation under external electric field stress.

Without electric field (52 Hbonds)			E = 0.001 V/nm (50 Hbonds)			E = 0.002 V/nm (54 Hbonds)		
Donor	Acceptor	Occupancy	Donor	Acceptor	Occupancy	Donor	Acceptor	Occupancy
GLY	GLU	5.14%	GLY	GLU	3.12%	GLY	GLU	11.86%
GLN	PRO	2.12%	GLN	PRO	8.08%	SER	GLU	21.30%
ASN	GLN	5.54%	GLU	SER	25.13%	GLN	PRO	3.10%
GLU	SER	9.50%	SER	GLU	40.05%	GLN	SER	24.04%
GLN-	SER	3.24%	GLU	GLU	6.08%	GLN	ASN	2.92%
GLN	ASN	3.44%	SER	GLY	2.02%	SER	ASN	2.38%
SER	GLU	22.00%	SER	GLY	8.26%	GLU	SER	2.02%
GLN	GLU	4.46%				SER	ASN	4.50%
SER	GLU	25.39%				GLU	SER	5.22%
GLU	GLU	9.78%						
SER	GLN	2.06%						

### 3.4. Radius of Gyration (Rg)

Another property that quantifies any change in the structure of protein is Rg. The Rg of the protein basically quantifies the distribution of the atoms in the space relative to their center of mass. It can be determined using Equation (3) [8].


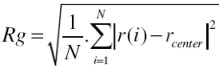
(3)
where *r*(*i*) is the coordinates of an atom *i* and *r_center _*is the coordinates of the protein’s center of mass, *N *is the number of atoms.

**Figure 6:**
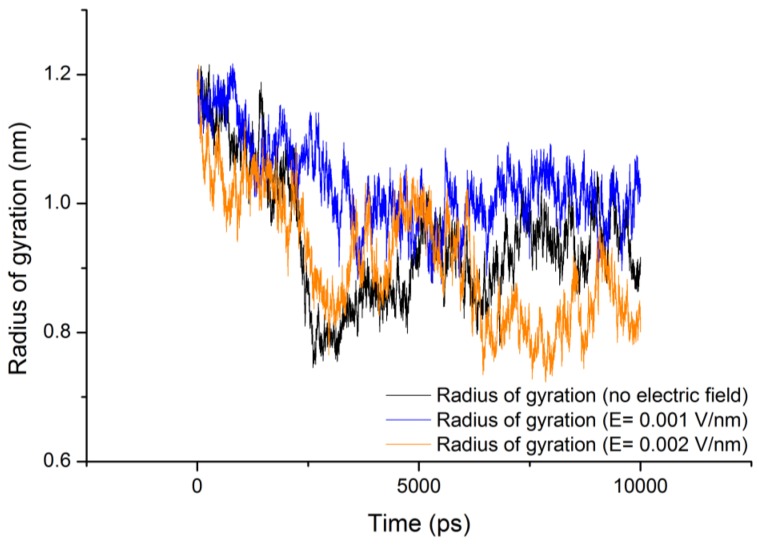
Radius of gyration (Rg) of gliadin protein under the stress of external electric field.

**Figure 7:**
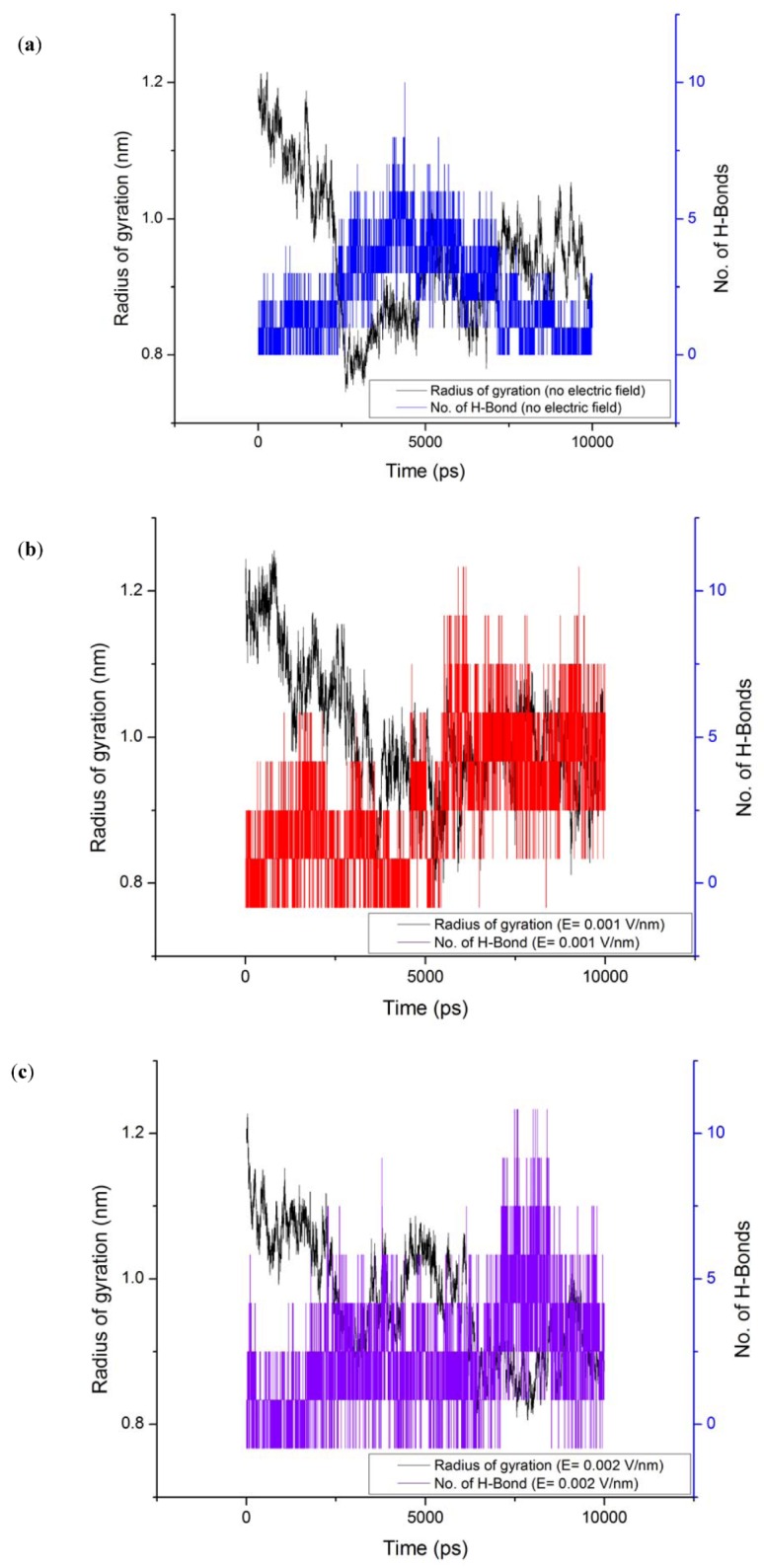
Radius of gyration evolution with respect to number of hydrogen bonds; (**a**) without electric field (**b**) 0.001V/nm; (**c**) 0.002 V/nm.

The radius of gyration (Rg) values for gliadin protein under the influence of electric field strength 0.001 V/nm and 0.002 V/nm were lower with respect to the reference (Table 2) (Figure 6), because under the influence of electric field it can be observed form Figure 7, that during the course of the simulation the protein tended to compact itself by forming turns via hydrogen bonds, For all the simulation conditions a common trend of decrease in radius of gyration was observed with increase in number of hydrogen bonds.

### 3.5. Solvent Accessible Surface Area (SASA)

**Figure 8:**
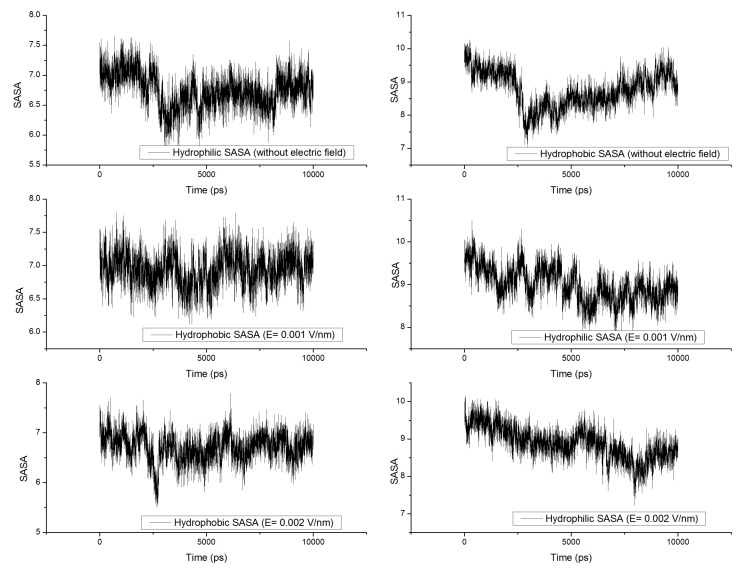
Changes in solvent accessible surface area [SASA (nm^2^)] under the influence of external electric field.

**Figure 9:**
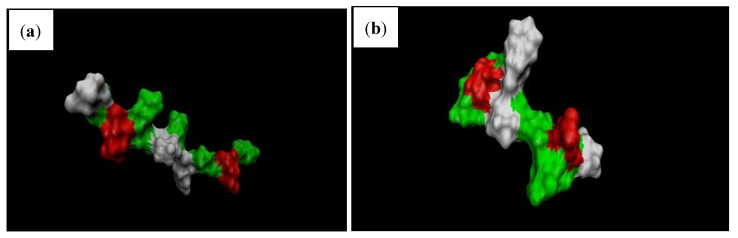
Snapshots of gliadin protein surface at the (**a**) beginning; and (**b**) end of simulation under external electric field strength of 0.002 V/nm. Note: non-polar residues (white), basic (blue), acidic (red) and polar residues (green).

Protein activity widely depends on its surface properties, which in turn depend on the structure of the protein (primary and secondary structures). Analysis of SASA provides information about proteins ability to interact with solvents and other molecules such as other proteins and enzymes [8]. It is known that when a protein is subjected to external stresses such as thermal, electrical or chemical, changes in structural configuration leads to varied surface properties [20]. In our study, no significant changes were observed in the solvent accessible surface area (Figure 8, Figure 9). Several researchers have observed changes in SASA over the period of the simulation under external electric field stress [8,10,21,22]; in our study, no variation was observed because the protein studied was too small with no defined significant secondary structure. Since α-β gliadin residues form mostly β-turns they become essential in enabling the gluten protein to fold and form compact tertiary structures, these structures minimize the proteins SASA. It is well known that turns are significant in governing the flexibility of regions in protein structure, which in turn affects its activity. Our results on RMSD and Rg of gliadin protein under the influence of high electric field suggest that decrease in their value leads to compactness of the gliadin protein structure, which may directly affect the overall tertiary structure of the gluten protein and its SASA. This would be interesting to investigate and further studies are required to prove this hypothesis.

## 4. Conclusions

The present study explored the effect of static external electric fields of strength 0.001 V/nm and 0.002 V/nm on the structural stability of gliadin protein. It showed that application of external electric field induced conformational changes in the protein by means of formation of hydrogen bonds between amino acid residues. It was also observed that formation of hydrogen bonds between residues during the course of simulation affected its root mean square deviation and radius of gyration values. Number of hydrogen bonds formed increased with an increase in electric field strength. We can also conclude that more work is required to analyze and evaluate the effect of external electric field stress on the protein structure and its functional properties, but the present study does demonstrate that electric field can be used to alter the structure of the protein, which will affect its functional properties. Knowledge gained through this MD simulation under static electric field will be useful to explore and explain the effect of novel food processing techniques such as microwave, radiofrequency, pulsed electric field and electrohydrodynamic drying on the biochemical composition of food products.
